# Analysis of Microbial Diversity and Evidence of Contamination at a Mars Analogue Habitat

**DOI:** 10.1111/1758-2229.70146

**Published:** 2025-07-06

**Authors:** Mara Leite, Dragana Dobrijevic, Michael C. Macey, Mamatha Maheshwarappa, John Ward, Lewis Dartnell

**Affiliations:** ^1^ School of Life Sciences University of Westminster London UK; ^2^ Department of Biochemical Engineering University College London London UK; ^3^ Faculty of Science, Technology, Engineering and Mathematics The Open University Milton Keynes UK; ^4^ QinetiQ Farnborough UK

**Keywords:** contamination, desert environment, human‐associated bacteria, Mars, Mars Desert research station, microbial diversity

## Abstract

We assessed the potential for a crewed mission to microbially‐contaminate the Martian surface by studying a terrestrial analogue facility, the Mars Desert Research Station. DNA sequencing of interior swab and exterior soil samples allowed characterisation of the microbiome present inside the habitation module and to seek evidence of escape into the desert surface outside the “airlock”. Microbial diversity of interior surfaces was dominated by Gram‐negative bacterial genera *Acinetobacter*, *Pseudomonas*, *Escherichia* and *Shigella*, with *Penicillium* and *Aureobasidium* most abundant amongst the fungal sequences—many members of which are human commensals. The soil microbiome sample was mostly characterised by Bacteroidota, Actinobacteriota and Pseudomonadota bacteria, with a number of extremophiles identified. The most abundant fungal genera were *Alternaria*, *Neocamarosporium* and *Preussia*. No archaeal sequences were isolated in either interior samples or soil. Principal Component Analysis of amplicon sequence variants shared between the soil sample and at least one indoor swab showed no evidence for contamination of the soil from the Hab microbiome. However, three bacterial genera—*Paracoccus*, *Cesiribacter* and *Psychrobacter*—identified in both soil and internal swabs are not commonly associated with humans and so represent evidence of backwards contamination of environmental microbes brought into the Hab during MDRS operations.

## Graphical abstract

We assessed the potential of a crewed mission to Mars to microbially contaminate the surface by studying a terrestrial analogue facility, the Mars Desert Research Station. DNA sequencing of interior swabs and an external soil sample allowed us to characterise the microbiomes present. We did not detect contamination of the desert soil within our detection thresholds, but did find evidence of backwards contamination of environmental microbes into the MDRS.

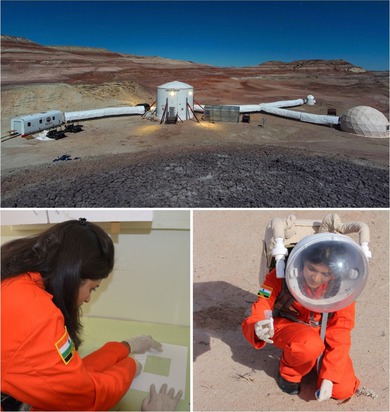

## 1 Introduction

Human‐associated microorganisms are often the source of environmental contamination in pristine and remote locations on our planet (Baker et al. 2003; Schuerger and Lee 2015; Sjöling and Cowan 2000; Upton et al. 1997). Within the context of human space exploration, such microbes carried by future astronauts to Mars could escape the pressurised habitats or other infrastructure and forward contaminate the Martian surface. No space vehicle or structure is perfectly airtight, allowing the potential leakage of the internal microbiome out into the external environment—particularly through airlocks or Extravehicular Activity (EVA). Such a possibility challenges planetary protection and would frustrate the efforts of astrobiology and the search for indigenous Martian life (Schuerger and Lee 2015). Of particular concern is the contamination of “special regions” – areas on Mars that could support the growth of terrestrial microorganisms or harbour extant life forms (Rummel et al. 2014). Even though current policies constrain robotic and future human missions from exploring these regions, it is necessary to better understand how human‐associated microbes may escape from the surface infrastructure of future surface missions and disperse into the Martian landscape (Schuerger and Lee 2015). Recently, Spry et al. (2024) identified key planetary protection knowledge gaps, including microbial monitoring of spacecraft and the potential for contamination of Mars by future crewed missions.

Human‐made structures (such as residential buildings, hospitals and the International Space Station) harbour complex microbial communities that are influenced by factors including environmental conditions, cleaning practises, building characteristics, and their occupants (Adams et al. 2015; Mayer et al. 2016; National Academies of Sciences, Engineering, and Medicine 2017). Indoor microbiomes can present hazards to built habitats and their residents (Blachowicz et al. 2017; Mayer et al. 2016; Mora et al. 2016); therefore, a growing number of studies have aimed to understand the main sources and routes of microbial dispersal and to provide insight into the spatial and temporal dynamics of indoor microbiota (Adams et al. 2015). Of particular relevance are the findings from microbial monitoring studies of the International Space Station and Mars/Moon analogue habitats (Novikova et al. 2006; Sielaff et al. 2019; Vesper et al. 2008).

Terrestrial analogue stations have been designed and constructed in extreme environments on Earth in an effort to emulate future crewed missions to Mars and space. Such analogue stations that have been subject to microbiome composition studies during simulated missions include the Hawaii Space Exploration Analogue (HI‐SEAS), the Concordia Antarctic base, the Inflatable Lunar/Mars Analogue Habitat (ILMAH), MARS500 and the Lunar Palace1 (LP1) (Blachowicz et al. 2017; Mahnert et al. 2021; Mayer et al. 2016; Schwendner et al. 2017; Van Houdt et al. 2012; Yang et al. 2022). For example, the surfaces of Mars500 were characterised by the presence of *Staphylococcus* and *Bacillus* (Schwendner et al. 2017), whereas monitoring of the HI‐SEAS interior environment revealed that *Chryseobacterium*, *Lactobacillus*, *Gardnerella*, *Prevotella* and *Acinetobacter* were the most frequent bacteria (Mahnert et al. 2021). Li et al. (2021) also investigated surfaces within the HI‐SEAS IV mission and observed significantly higher microbial diversity on plastic rather than wooden surfaces. Mayer et al. (2016) reported an increase of *Actinobacteriota* and *Bacilliota* phyla over time during the ILMAH 30‐day mission.

**FIGURE 1:**
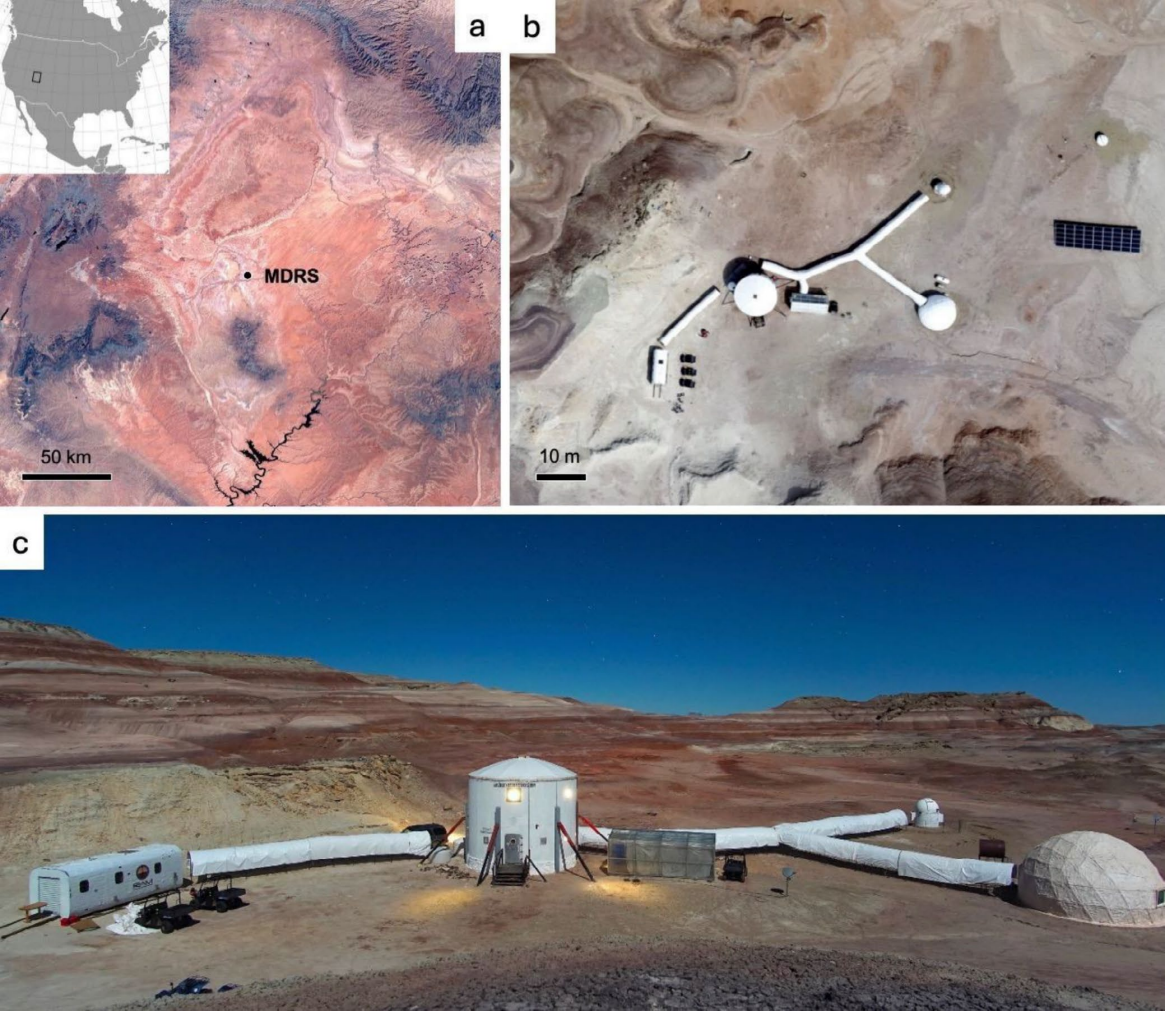
The Mars Desert research station (MDRS) near Hanksville, Utah, USA: (a) Regional view showing location within the Utah desert; (b) aerial view of the site; (c) layout of the MDRS facility, showing, from left to right: the Repair and Assembly Module (RAM), the Hab (the tallest building), The GreenHab, the observatory, and the Science Dome. (Image credits: (a) Created using Mathematica 12.0; (b) The Mars Society; (c) P.C. Sokoloff, reproduced under Creative Commons Attribution 4.0 International licence).

Here, we conducted a sampling and DNA sequencing study on the Mars Desert Research Station (MDRS), constructed and operated by the Mars Society and located near the small town of Hanksville, Utah, USA (Figure 1). The Utah desert is a shale desert, rather than a sand desert, with mineralogy comparable to that of the martian surface. The soil surrounding the MDRS consists of sedimentary deposits of silicates, phylosilicates (clays), evaporites and is characterised by red‐coloured iron oxides (Direito et al. 2011). The MDRS includes the habitational module (Hab), a greenhouse (GreenHab), a repair and assembly module (RAM), a laboratory (The Science Dome), and an observatory (Figure 1c). It is a living and working station where seven crew members remain in confinement for the entire duration of their mission. We firstly characterised the internal microbiome of the Hab module dominated by the human presence, and secondly analysed a soil sample from immediately outside the Hab “airlock” to attempt to detect evidence of such human‐associated microbes contaminating the external environment.

## 2 Materials and Methods

### 2.1 Sample Collection

**FIGURE 2:**
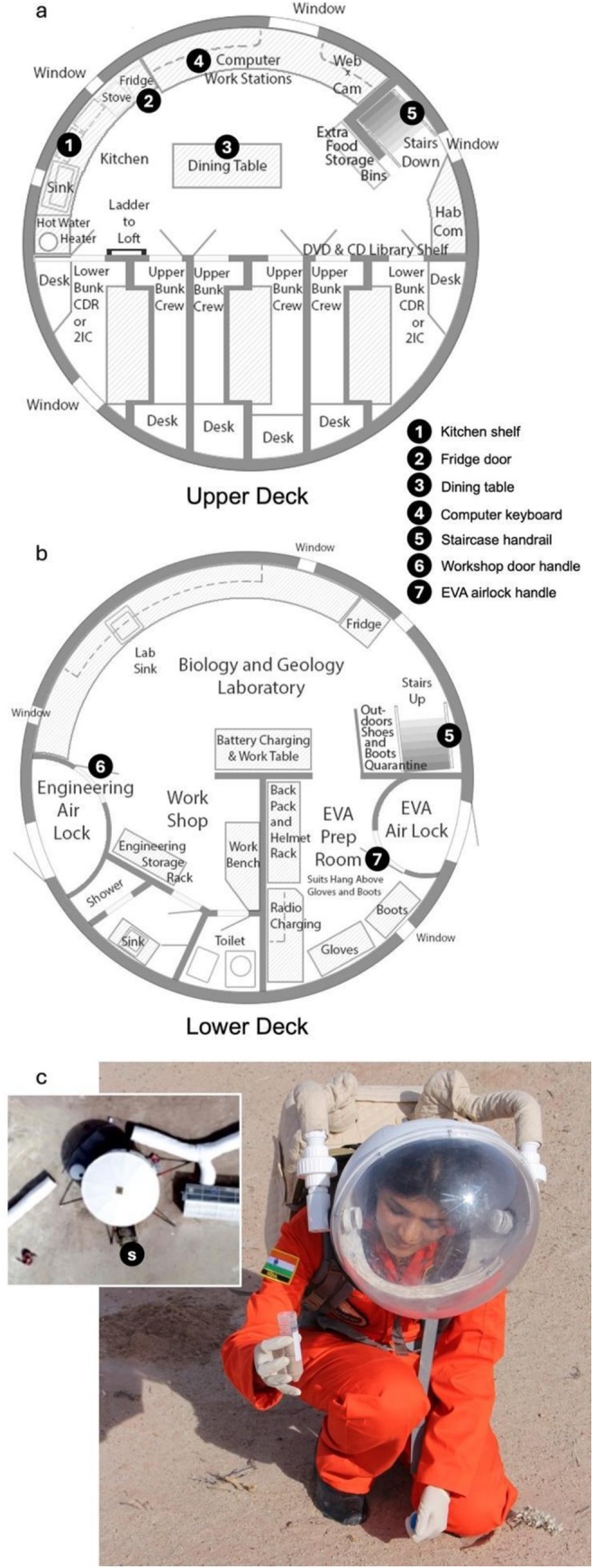
Sample collection locations. The interior layout of the Hab module, indicating the swab surface sampling locations of (a) the upper deck and (b) the lower deck. (c) Sampling of soil immediately outside the Hab airlock (inset) by co‐author Maheshwarappa. Swab location diagrams based on floor plans drawn by Hugh S. Gregory, MDRS Document Editor.

All samples were collected in and around the MDRS Hab module at the end of the crew's two‐week mission in February 2017 (MDRS mission 174). Swab samples from interior surfaces were collected in duplicate before end‐of‐mission cleaning, from locations shown in Figure 2. Four swab samples were collected from the upper (habitational) deck: kitchen shelf (1); fridge door (2); dining table (3); and computer keyboard (4). Three swab samples were collected from the lower deck: workshop door handle (6); the interior handle of the EVA “air lock” that gives access to the exterior (7); and the staircase handrail (5) that connects both decks. We therefore targeted sampling to dry, high‐touch surfaces (HTS) such as handles, handrails and keyboards, as well as kitchen areas, as recommended in the literature (National Academies of Sciences, Engineering, and Medicine 2017). Whilst wearing ethanol‐sterilised latex gloves, sterile swab kits (FloqSwabs, Copan, Italy) were moistened in sterile Phosphate‐Buffered Saline (PBS) and run across the surfaces. For large, flat, surfaces, such as the dining table, a 10 × 10 cm surface area was swabbed in horizontal, vertical and diagonal directions. For non‐planar surfaces, such as door handles, it was attempted to sample an approximate 10 × 10 cm area, using as much of the surface of the object as possible. The swab was rubbed and rotated against the surface to ensure maximum microbial transfer. After sampling, swabs were returned to their original container, sealed with tape, and placed in individual polyethylene bags. A triplicate soil sample for assessing microbial leakage from the Hab was collected immediately adjacent to the habitat “airlock” door by a crew member dressed in full (simulated) EVA suit with ethanol‐sterilised gloves scooping the soil into sterile 50 mL Falcon tubes (Figure 2c). During this mission, one EVA was conducted per day through this (non‐pressurised) airlock. All samples were refrigerated at 4°C before departure from the MDRS and then stored in the lab at −80°C until they were analysed.

### 2.2 DNA Extraction and Sequencing

Two millilitres of sterile PBS was added to each swab container, and then agitated for 10 min at maximum speed (250 rpm) inside an incubator. The PBS was transferred into a sterile Eppendorf microfuge tube and centrifuged at 20,000 g for 10 min. The supernatant was removed without disturbing the pellet and transferred back to the swab container to be agitated for another 10 min. After a second centrifuge step, the pellet was resuspended in the C1 lysis buffer from the DNeasy PowerSoil Kit (Qiagen) and transferred to the bead beating tubes. The kit manufacturer's instructions were followed for all subsequent steps. Swab DNA concentrations ranged between 0.3 and 6.4 ng/μL. Twenty grams of soil was used for the total DNA extraction with DNeasy PowerMax Soil Kit (Qiagen) following the manufacturer's instructions. DNA quality check, library generation, and sequencing on Illumina MiSeq platform was performed by Eurofins Genomics as part of their INVIEW Microbiome Profiling 3.0 service. Three targets were amplified: bacterial V3–V4 hypervariable region of the 16S rRNA gene, the entire archaeal 16S rRNA gene, and the fungal internal transcribed spacer gene, ITS1.

We also analysed a negative control “blank” to characterise the kitome present in this study. The PBS used to moisten the sample‐collection swabs in the field was added to an unexposed swab kit. Following the same protocol as that used for the samples, the PBS was subjected to DNA extraction and ITS and 16S rRNA gene sequencing to enable the identification of any contaminant DNA present in the swabs, PBS or reagents in the Qiagen DNA extraction kits.

### 2.3 Bioinformatics and Statistical Analysis

The raw sequencing data was processed using the QIIME2 pipeline (Bolyen et al. 2019). The amplicons were demultiplexed and primers and barcodes removed from all reads. The DADA2 noise removal algorithm was used to remove all chimeric sequences and the first 15 bp of all sequences. ITS and 16S rRNA gene amplicons were trimmed based on the Phred values (fungal ITS amplicons *F* = 260 bp and *R* = 230 bp; prokaryotic16S rRNA gene *F* = 270 bp *R* = 235 bp). Sequences were then clustered into amplicon sequence variants (ASVs) using the DADA2 algorithm. Phylogeny was assigned to the amplicon sequence variants using Scikit–learn classifier (Pedregosa et al. 2011), which compared the ASVs against the Silva (for bacterial and archaeal 16S rRNA gene amplicons) and UNITE (for fungal ITS amplicons) databases (Kõljalg et al. 2020; Nilsson et al. 2019; Quast et al. 2013; Yilmaz et al. 2014) with a confidence threshold of *p* = 0.7. The ASVs were aligned using MAFFT (Katoh and Standley 2013) and a rooted tree produced. All of the amplicons were normalised by rarefaction to 60,000 reads and alpha and beta diversity metrics calculated from the normalised data using QIIME2 (Bolyen et al. 2019) to assess the community diversity and the variation between samples. The alpha diversity metrics used were Shannon, Simpson and Chao, and the beta diversity metric was Bray–Curtis dissimilarity index. Principal component analysis was performed using ClustVis (Metsalu and Vilo 2015). The data was processed and analysed using R (version 4.0.2) and the tidyverse and readr packages (R Core Team 2020; Wickham et al. 2019, 2024). The Shapiro–Wilk test (Shapiro and Wilk 1965) was used to assess the normality of the abundance data for each location. Given the non‐normal distribution of the data, the Kruskal‐Wallis test (Kruskal and Wallis 1952), a non‐parametric method, was used to compare the abundance distributions of bacteria and fungi between different locations.

## 3 Results and Discussion

In order to characterise the interior microbiome of an analogue Martian crew habitat, seven swab samples were collected from frequently touched surfaces within the MDRS Hab (Figure 1a,b): (1) kitchen shelf, (2) fridge door, (3) dining table, (4) computer keyboard, (5) staircase handrail, (6) workshop door handle, (7) interior handle of the EVA air lock. A soil sample was also collected immediately outside the habitat airlock in order to assess the potential detectability of human‐associated microbes from the Hab contaminating the environment.

We'll first discuss here our investigations of the background sequences contained in the molecular biology kits used, the microbiome characterised within the MDRS Hab, then focus on human‐associated genera in the internal environment, before finally examining evidence for contamination of the external environment by human commensal microbes.

Sequence analysis identified bacteria and fungi in all swab samples taken from surfaces inside the habitat, with the exception of the staircase handrail where only fungal sequences were amplified. The number of sequence reads was generally higher for fungi (Figure SI1). Archaeal sequences were not amplified from any of the samples. Similar studies have attributed the difficulty in detecting archaea from surface sampling to differences in environmental distribution: archaea tend to inhabit microhabitats, whilst bacteria are more broadly distributed in the environment (Aller and Kemp 2008; Direito et al. 2011). The role of archaea in the microbiome of human‐built environments is still unclear due to the lack of sufficient archaeal surveys (Moissl‐Eichinger 2011; Mahnert et al. 2019). Nonetheless, screening for archaea on spacecraft and analogue habitat surfaces remains essential as they are considered to possess metabolisms that may enable survival and proliferation in Martian conditions (Moissl‐Eichinger 2011).

### 3.1 Investigating the “Kitome”

One of the challenges in environmental microbiota research is the presence of contaminating DNA in extraction kits (Salter et al. 2014). It is now well‐established that kits and reagents contain their own microbiome (“kitomes”) which can mask the microbiome of samples (de Goffau et al. 2018; Kim et al. 2017; Hornung et al. 2019; Stinson et al. 2019; Salter et al. 2014; Velásquez‐Mejía et al. 2018). The presence of kitomes, which varies between kits, is particularly problematic for the analysis of samples containing low microbial biomass (de Goffau et al. 2018; Salter et al. 2014; Stinson et al. 2019), such as desert soil. In such samples, the amount of DNA of interest might not be enough to compete with the contaminating DNA (Salter et al. 2014).

Concerns regarding the lack of negative controls in microbiome and kitome research have also emerged in recent years (Hornung et al. 2019). Previous studies have reported the presence of contaminating DNA in PCR reagents, kits, and even molecular biology grade water (Hornung et al. 2019; Kim et al. 2017; Kulakov et al. 2002; Salter et al. 2014; Stinson et al. 2019; Velásquez‐Mejía et al. 2018). These kit contaminants match the sequences of soil‐ and water‐associated bacteria, including nitrogen fixers (de Goffau et al. 2019; Kulakov et al. 2002). A strength of our approach is the use of a blank to characterise the kitome present in this study. Our kitome sequencing results suggest contamination of our indoor samples with soil and root‐associated bacteria such as *Bradyrhizobium*, *Mesorhizobium* and *Phyllobacterium* (Table SI1), many of which fix nitrogen and have been previously reported as kitome contaminants (Salter et al. 2014): *Bradyrhizobium* is one of the most common contaminants in sequencing datasets (Laurence et al. 2014; Salter et al. 2014). A possible explanation for the presence of nitrogen‐fixing bacteria in kitomes is the use of nitrogen instead of air during storage of ultrapure water (Kulakov et al. 2002; Salter et al. 2014). These contaminant taxa are commonly associated with plants or soils, and therefore their exclusion from further analysis will not preclude the detection of forward contamination of human commensal microbes from the MDRS Hab interior into the environment. Fungal contamination was also evident in our study of the kitome. The majority of contaminating sequences identified in the negative control belong to saprotrophic genera, predominantly *Leptobacillium* and *Exophiala*, which were also present in high numbers in the indoor environment (Table SI2).

Whilst it is practically impossible to eliminate kitomes altogether, the inclusion of controls and careful attention to sample collection and processing are common procedures used to minimise contamination risks (de Goffau et al. 2019; Hornung et al. 2019; Salter et al. 2014). Beyond these preventive measures, bioinformatics tools can also be used to detect ASVs shared with the negative controls and sample data (Edmonds and Williams 2017; Hornung et al. 2019). Currently, no standard protocol exists for interpretation of 16S rRNA sequencing controls and raw data (Hornung et al. 2019). The elimination of the kitome from all samples is a possibility but should be carefully considered and take into account the number of reads obtained; to exclude suspected contamination from sample results, the negative control should have fewer reads than the samples (Edmonds and Williams 2017; Hornung et al. 2019).

In this study, kit contaminants were studied at the genus level. The number of ASVs identified in the control was compared with those from the samples. If a certain genus was recovered in a sample and the number of reads was higher in the negative control, that genus was removed from the microbiome analysis. We found bacterial and fungal contaminant sequences were almost entirely absent from the soil sample (Tables SI1 and 2), suggesting that the source of the kitome was the swab kits and/or PBS used to sample surfaces within the Hab, rather than the DNA extraction kit.

### 3.2 Overview of Microbial Taxa Identified Across Surfaces Inside the Hab

**FIGURE 3:**
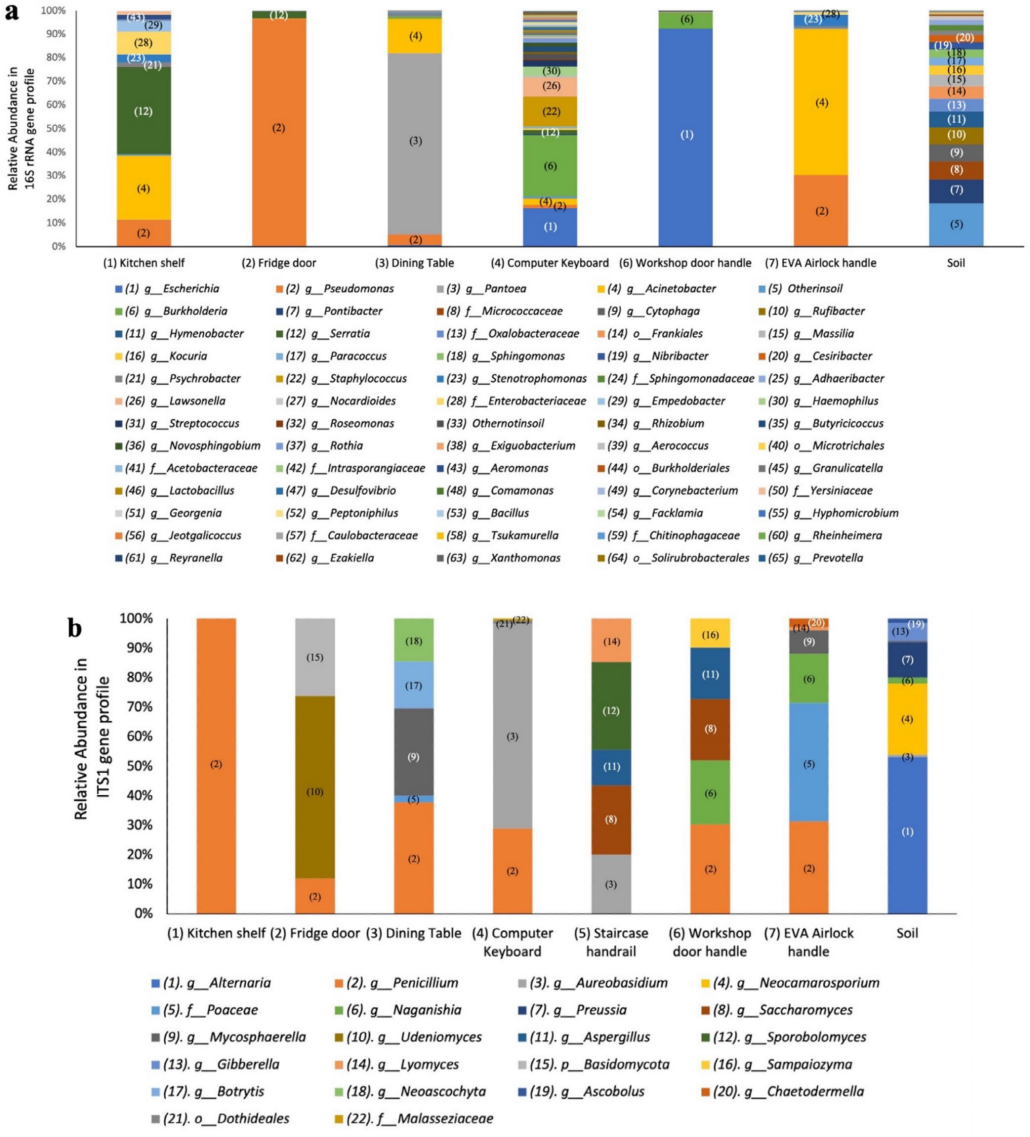
Composition of the sampled microbiomes shown as stacked column charts of relative abundance percentages, for (a) bacterial and (b) fungal sequences. Sequences are identified mostly to the genus level, with some exceptions where the genus could not be determined and the microbes were assigned to a higher classification level (e.g., family or order).

Once the kitome contaminant ASVs were identified and excluded, we calculated the relative abundances (percentage) of the most common microorganisms in all samples. Sequences were generally classified to genus level, but where taxonomic identity could not be resolved to genus, ASVs were assigned to a higher hierarchical classification level, such as family. Stacked column charts of these identified bacteria and fungi were constructed to facilitate visualisation of the sampled taxa, as shown in Figure 3.

As is clear from this plot, at the genus level the most common bacteria found inside the Hab were Gram‐negative *Acinetobacter*, *Pseudomonas* and *Escherichia*. No bacterial sequences were amplified from the swab sample of the staircase handrail (5), and so this sampling site is not shown in Figure 3a.

Almost half (48.8%) of the bacteria identified in the kitchen shelf (1) belonged to the *Acinetobacter* genus, a common skin‐associated bacterium (Mahnert et al. 2021) and will be discussed further. Curiously, the kitchen shelf had the lowest number of bacterial ASV reads (Figure SI1), possibly due to the cleaning regime. If the kitchen shelf is cleansed between MDRS crew missions and then touched directly less often than the dining table, for example, during inhabitation, it will experience less accumulation of bacteria.

Representatives of the genus *Pantoea* made up the largest portion (78.0%) of the dining table surface (3) microbiome. Members of this genus, and other representatives of the *Erwiniaceae* family, are commonly associated with plants and include a number of plant pathogens (Cruz et al. 2007; Llop 2015), and recent studies have reported the presence of *Pantoea* in various clinical isolates (Soutar and Stavrinides, 2019; Walterson and Stavrinides 2015). *Erwiniaceae* sequences were not significantly represented in the other Hab sampling sites (nor in the desert soil sample), suggesting that the members of this family detected on the dining table constitute a microbial fingerprint of fresh fruits and vegetables being prepared for consumption.

By comparison, the fridge door (2) exhibited very low bacterial diversity; 99% of reads in this sample were assigned to *Pseudomonas*. Members of this genus include human commensals and pathogens and are widespread in environmental niches, and also include psychrotrophic and psychrotolerant bacteria responsible for the spoilage of refrigerated food (Mahnert et al. 2021; Franzetti and Scarpeluni 2007).

The highest bacteria diversity of all the internal Hab sampling sites was observed on the keyboard (4) (Simpson −2.43, Shannon—0.80, Chao—65), with the closest in diversity being the Kitchen Table (Simpson—1.78, Shannon—0.76) (Table SI3). This is likely a consequence of this computer device, compared to other sampling sites, experiencing a very high rate of touching coupled with being rarely subjected to cleaning. The dominant genera identified on the keyboard were *Burkholderia* (35%), *Escherichia* (22%), and *Staphylococcus* (17%) with 14 other genera present at more than 1% relative abundance. Similarly, whilst studying intensive care units, Bures et al. (2000) concluded that the incidence of novel and unrecognised taxa was greater on keyboards and faucet handles than on other well‐studied ICU surfaces.

Intriguingly, the ITS amplicons detected numerous sequences identified as Viridiplantae rather than fungi across the indoor environments (except for the staircase). These included multiple types of vegetables and herbs, which could have originated from either the food supplied to the analogue astronauts or the plants grown in the MDRS greenhouse. These plant sequences represented 0.3%–10.1% relative abundance in the ITS profiles. The genus *Poaceae*, which includes cereal grasses, was identified in significant quantities on the Kitchen shelf, Fridge door and EVA door handle, with the highest abundance on the EVA door handle (19,052 reads). Additionally, reads of the genus *Allium* (onion) were present on the Fridge door and EVA door handle, and the handle also contained *Solanum* (potatoes and tomatoes). The Fridge door ITS profile was especially rich in *Fabaceae* family members, including *Vigna* (cowpea), *Vicia* (vetch), *Glycine* (soybean) and *Cicer* (chickpea), as well as other plants such as *Daucus* (carrot) and *Cuminum* (cumin). The top of the dining table had lower diversity, but included *Glycine* (soybean) and *Camellia* (tea). The Keyboard ITS profile contained reads identified as *Urtica* (nettle), *Brassica* (cabbage) and *Coriandrum* (coriander). We also detected *Lawsonia* (0.5%) on the keyboard. This plant is the source of the henna dye often used for decorative staining of the hands, feet and hair. Consequently, the presence of *Lawsonia* in this location might be attributed to the presence of henna dye on crew members' fingertips as they typed on the keyboard.

Only members of the *Enterobacteriaceae* family (99.9%) were identified on the workshop door handle (6). This family includes human‐associated commensals and pathogens such as the closely related *Escherichia* and *Shigella* genera (Schierack et al. 2007). In a recent study, members from these genera were identified as the most common Gram‐negative bacteria isolated from frequently touched hospital surfaces, including door handles (Bhatta et al. 2018). Additionally, this was the sample with the highest bacterial ASVs reads (Figure SI1). Analysis of the interior handle of the EVA air lock (7), the sample closest to the exterior, revealed the presence of *Acinetobacter* (62.3%) and *Pseudomonas* (30.3%).

Overall, this study identifies Pseudomonadota as the most abundant bacterial taxon present in all sampling sites inside the MDRS habitational module. Whilst we did not attempt to collect samples before inhabitation, and so do not have temporal data, the predominance of proteobacterial species on all sampled surfaces at the end of the mission may be the result of a loss of microbial diversity and a shift in the microbial composition from Gram‐positive to Gram‐negative bacteria. Previous studies have found that increased confinement and cleaning is associated with a shift from Gram‐positive towards Pseudomonadota and other Gram‐negative bacteria (Mahnert et al. 2019). Studies similar to this present one, specifically focused on space‐like habitats, have found a change in the microbiota as a result of human presence (Blachowicz et al. 2017; Schwendner et al. 2017). Confinement has usually been found to correlate with loss of microbial diversity (Schwendner et al. 2017), but more recently, Mahnert et al. (2021) observed a (slight) diversity increase in HI‐SEAS built surfaces.

Characterisation of the interior mycobiome (Figure 3b; Table SI4) is also crucial: not only are some fungal species potentially harmful to humans, technophilic fungi are capable of corroding structural materials and lead to habitat deterioration (Blachowicz et al. 2017; Mora et al. 2016; Novikova et al. 2006). A Bray–Curtis dissimilarity matrix was calculated to assess the pairwise dissimilarity between samples. Based on the matrix, the sample most similar to the soil sample was identified as the EVA door handle with a dissimilarity value of 0.967, possibly due to contact with crew members' hands on return from external activities. Ascomycota, the largest phylum of fungi, was abundant in all our swab samples (Figure SI3). Similarly, Blachowicz et al. (2017) report Ascomycota as the predominant phylum inside the Inflatable Lunar/Mars Analogue Habitat (90% of all characterised ASVs). On a genus level, as shown in Figure 3b, the predominant genera we detected through the ITS1 region were *Penicillium*, seen in all seven swabs, followed by *Aureobasidium*. Novikova et al. (2006) also report *Penicillium* (and *Aspergillus*) as the dominant fungal population aboard the ISS, whereas inside the inflatable Luna/Mars analogue, an increase in mycobiome diversity was recorded over the 30‐day mission, but with members of the *Pleosporaceae* family remaining dominant (and specifically of the *Epicoccum* and *Alternaria* genera) (Blachowicz et al. 2017).

*Mycosphaerella* fungal genus was found on the top of the dining table, probably due to food preparation in the kitchen and this genus' presence as a plant‐associated fungus. *Mycosphaerella* is one of the largest genera of plant pathogenic species and is responsible for causing, for example, serious leaf spot diseases in banana leaves (Zeng et al. 2017; Arzanlou et al. 2010). *Udeniomyces* is another plant‐associated fungal genus identified in abundance on the fridge door (62%). Species from this genus have been recovered from the leaves of plants, indicating its potential as an endophyte (Niwata et al. 2002).

### 3.3 Human‐Associated Microbes Inside the Hab

The astronauts themselves are the most important sources of contamination in space habitats (Novikova et al. 2006; Schwendner et al. 2017; Yang et al. 2022). Commensal microbes that inhabit multiple parts of the human body, such as skin or respiratory airways, become dispersed via skin flakes, perspiration, or coughing, and consequently shape the interior microbiome (Baker et al. 2003; Blachowicz et al. 2017; Novikova et al. 2006).

Numerous genera can be indicative of human microflorae. The predominant cutaneous residents are Gram‐positive bacteria from the *Staphylococcus*, *Streptococcus, Corynebacterium*, *Propionibacterium and Brevibacterium* genera (Cogen et al. 2008; Cosseau et al. 2016). Within the Gram‐negative bacteria, *Acinetobacter* is most frequently isolated from skin microflora (Cosseau et al. 2016), whereas coliform bacteria are common representatives of the human gastrointestinal tract and include genera from the *Enterobacteriaceae* family (such as *Escherichia* and *Shigella*).

In the present study, enteric bacteria were traced to all indoor locations (Figure 3a). The EVA door handle and the fridge door had the lowest relative abundances, which may reflect a more thorough cleaning schedule and hygiene practises in these areas. Additionally, the EVA door handle might be more likely to be touched only when wearing gloves. In contrast, a significant number of *Enterobacteriaceae* were found in the keyboard (60.2%) and kitchen shelf (17.4%). Members of the *Escherichia–Shigella* genera were highly abundant on the workshop handle (> 90%).

Sequences from skin‐associated *Staphylococcus* and *Streptococcus* were seen in the keyboard, which could be the result of frequent and direct touch from crewmembers. ASVs assigned to *Acinetobacter* were present in all indoor samples to varying proportions. This genus is recognised as a skin taxon and is also frequently isolated from human‐made surfaces, including indoor spaces with strictly controlled conditions such as cleanrooms (Mahnert et al. 2019, 2021). Mahnert et al. (2021) observed a higher abundance of *Acinetobacter* in the HI‐SEAS built surfaces (e.g., main room and bedroom) than on the crew's skin. This finding made the authors consider the potential role of this bacterium as a microbial indicator for confined human‐built environments. Members of the phyla Bacilliota and Bacteroides, which represent more than 90% of the human gut bacteria (Kosiewicz et al. 2011), were also present (Figure SI2).

In addition to bacteria, many fungi have also been identified as human commensals (Auchtung et al. 2018; Limon et al. 2017). *Penicillium* was the most common fungi inside the Hab, found in all sampled surfaces, in the following order of abundance: kitchen shelf (100%), top of dining table (37.8%), EVA door handle (31.3%), workshop door handle (30%), keyboard (28.9%), fridge door (11.9%), and staircase handrail (< 1%). The presence of *Penicillium* in healthy human stools is documented (Limon et al. 2017).

The *Saccharomyces* fungal genus was recovered from the staircase (23.54%) and the workshop door handle (20.71%), and has been previously reported as a prevalent member of indoor fungal communities, including interior air and dust samples (Estensmo et al. 2022, 2021; Martin‐Sanchez et al. 2021) suggesting a commensal relationship with the human habitat. Furthermore, *Saccharomyces cerevisiae* , prominent in food production, is also a commensal fungus abundant in the human gut of healthy individuals (Martin‐Sanchez et al. 2021; Paterson and Underhill 2017).

The *Naganishia* fungal genus was relatively abundant in both the EVA (16.6%) and workshop door handles (21.7%). Species from this genus exhibit extremophilic characteristics (e.g., resistance to UV, extreme dryness and low nutrient availability) and have been identified in diverse and extreme environments with Mars‐like characteristics, such as the Atacama desert and the Dry Valleys of Antarctica (Schmidt et al. 2017). *Naganishia* representatives have also been identified on human skin, gut, scalp and oral cavity (Li et al. 2021), in the gastrointestinal tract of pigs (Li et al. 2021) and in indoor environments, demonstrating its commensal nature (Timm et al. 2020), and sometimes also as an opportunistic pathogen (Oliveira et al. 2023). These findings demonstrate that the *Naganishia* fungal genus is capable of surviving in extreme environments whilst also exhibiting a ubiquitous and opportunistic presence in association with humans and other animals.

Human skin and the oral cavity commonly harbour *Aspergillus* (Limon et al. 2017), which was present in the workshop door handle and staircase handrail. *Aureobasidium* is a genus frequently encountered in healthy oral mycobiota (Underhill and Iliev 2014), and sequences assigned to this genus were recovered from the staircase and keyboard.

Moreover, a handful of bacterial and fungal genera found inside the Hab include potential human pathogens and opportunists: *Pseudomonas*, *Escherichia*, *Aspergillus* and *Aureobasidium* all contain species known to cause infectious diseases in humans (Limon et al. 2017; Mora et al. 2016). These findings emphasise the necessity to monitor the microflora of confined environments in analogue and future Mars missions to avoid the spreading of harmful microorganisms. Confined conditions can aggravate bioaccumulation and microbial transmission due to increased physical proximity amongst occupants; furthermore, spaceflight can compromise immune responses whilst enhancing antibiotic resistance and bacterial virulence of some microbial species (Blachowicz et al. 2017; Mahnert et al. 2021; Mayer et al. 2016; Mora et al. 2016; Schwendner et al. 2017; Yang et al. 2022).

### 3.4 Microbial Taxa Identified in the Soil

We also performed analysis on a soil sample collected immediately outside the MDRS Hab airlock to characterise the environmental microbiological community and assess if contamination by human commensal microbes was detectable.

Figure 3 shows the soil microbial community to be a great deal more diverse than the Hab interior microbiome. Most of the bacteria identified in the soil belonged to the phyla: Bacteroides, Actinobacteriota and Pseudomonadota (Figure SI2). Members of these phyla have been found in deserts worldwide, including MDRS soil samples from past studies (Direito et al. 2011; Sun, Shi, et al. 2018). In this study, we identified *Pontibacter* (8.9%), *Cytophaga* (6.5%), *Rubibacter* (6.3%) and *Hymenobacter* (6.1%) as the most abundant genera—members of which are often identified in both soil and aqueous environments (Kirchman et al. 2003; Roiko et al. 2017; Sun, Xing, et al. 2018).

Of particular interest within astrobiology is the presence of extremophilic microbes, which have implications for the origin of life on Earth and the search for microbes on other planetary bodies (Merino et al. 2019). Deserts are extreme locales characterised by environmental stressors that limit microbial survival (Sun, Shi, et al. 2018; Vikram et al. 2016). The MDRS is located in a Martian analogue environment: a cold desert with an average annual temperature of 12°C and wide diurnal temperature variations (minimum and maximum recorded temperatures of −36°C and 46°C), respectively (Direito et al. 2011; Vikram et al. 2016). Whilst it is not possible to assess the metabolisms of the organisms detected in this study solely via 16S rRNA gene sequencing, the community profiles did contain taxa associated with extremophilic adaptations. Amongst these taxa identified was the psychrophilic species *Hymenobacter glacialis* (Roldán et al. 2020) and other cold‐loving bacteria such as *Planomicrobium glaciei* and *Psychrobacter cryohalolentis* . Sequences assigned to the halotolerant species *Anditalea andensis* were also present. We also detected sequences identified as closely related to microbes associated with UV tolerance (e.g., *Rufibacter tibetensis*) as well as genera such as *Deinococcus* that are able to tolerate not only high levels of desiccation but also ionising radiation.

In our analysis of the fungal diversity within the soil sample, Ascomycota was found to be the predominant phylum (Figure SI3). At the genus level, the taxa detected by ITS1, in order of abundance, were: *Alternaria* (48.7%), *Neocamarosporium* (21.8%) and *Preussia* (11%). *Alternaria* is commonly detected in cryptogamic soils (Bhatnagar and Bhatnagar 2005)—desert soils with a fragile surface crust characterised by the presence of cyanobacteria and lichen. Both *Alternaria* and *Preussia* are also frequently isolated from the soil and decaying vegetation (Gonzalez‐Menendez et al. 2017; Thomma 2003). *Neocamarosporium* species, which are typically halotolerant and distributed in saline regions (Gonçalves et al. 2019), were also recovered. This finding is in accordance with Bhatnagar and Bhatnagar (2005), who observed the presence of halophiles and haloalkaliphiles in desert crusts. Direito et al. (2011) also studied the microbiome of soil by the MDRS and reported a higher abundance and diversity of bacteria when compared to fungi. Similar to our study, no archaea were detected in their soil sample collected closest to the station.

### 3.5 Comparison Between the Indoor and Outdoor‐Derived Microbiomes

Monitoring pristine environments for microbial contamination as a result of human activity is essential for mitigating environmental degradation and for improving practises that can help reduce the risk of further contamination (Baker et al. 2003). For Mars, future crewed missions risk contaminating the planetary environment with human‐associated bacteria or other non‐indigenous microbes (including extremophiles), and so could potentially frustrate the differentiation between any indigenous microbes and contaminants and thus the search for life on Mars (Schuerger and Lee 2015; Yair et al. 2021). Although extreme conditions may limit the viability of human‐associated microbes on the Martian surface or in Mars analogue environments on Earth, plausible contamination in analogue locations has been reported in places such as the vicinity of camp sites in Antarctica (Baker et al. 2003; Sjöling and Cowan 2000), during a rover traverse in the Arctic (Schuerger and Lee 2015), and more recently in Israel's Ramon crater (Yair et al. 2021). The central aim of this present study was to investigate the potential for human‐associated microbes to leak out of the MDRS Martian analogue habitat and contaminate the surrounding desert soil.

As discussed above, sequence analysis of the sampled microbial communities shows that the abundance patterns of bacterial and fungal diversity of the MDRS interior surfaces do not resemble those of the external environment.

The stacked column charts in Figure 3 make clear that whilst the relative abundance patterns are distinct for each internal swab sample, reflecting differences in their history of human contact and cleaning, as well as the microenvironment of the locations, there is nonetheless a great deal of overlap in the microbial groups present. This indicates, similarly to the conclusions of Schwendner et al. (2017) studying the Mars500 habitat, that there is a common microbial signature inside the Hab even though each surface had its own microflora. In contrast, the microbiome of the soil sample is a great deal more diverse and contains many microbial groups not identified in any of the internal swabs. The most abundant bacterial genera found in the soil were absent in all seven indoor samples. The same trend was observed for fungi: 28 fungal genera were exclusively found in the soil.

**FIGURE 4:**
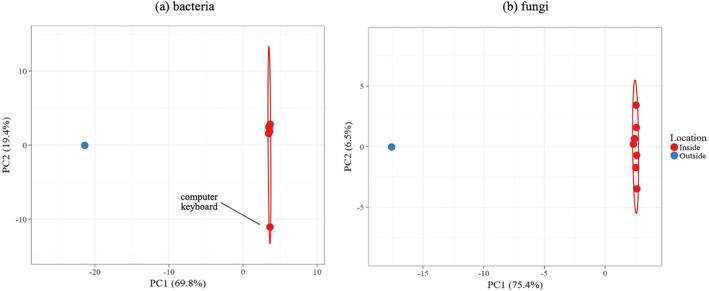
Principal coordinate analysis (PcoA) of dissimilarities between samples collected from the Hab (red) and soil (blue) for (a) bacteria and (b) fungi.

The potential contamination of the environment outside the Hab by human‐associated microbes was assessed by Principal Coordinate Analysis (PCoA). The plots shown in Figure 4 were constructed by comparing amplicon sequence variants (ASVs) shared with the soil sample and at least one indoor swab. The microbiome distribution of the overlapping samples was separated into two different clusters. Figure 4 shows the visible differentiation between internal swabs and soil for both bacteria and fungi. Indoor samples (red) clustered together. The outlier in the bacteria PCoA plot (Figure 4a) is the keyboard swab sample. The outdoor environment (shown in blue) was separated from the indoor sample data points along PC1. The significant difference between the indoor and outdoor fungal and bacterial profiles was confirmed with Kruskal Wallis analysis (*p* < 0.05 for both bacterial and fungal data). These results are consistent with the absence of significant contamination of the soil by taxa from inside the Hab.

At the genus level, based on the high abundance of *Pseudomonas* in many internal environments (Kitchen shelf, Fridge door, Keyboard and EVA door handle), its detection in the soil bacterial profile, and the fact that some members of this genus are human‐associated, this genus was identified as a potential instance of forward contamination. However, analysis of the bacterial diversity at the ASV level identified the internal and external populations of *Pseudomonas* as distinct and not indicative of transference. At the ASV level, we identified three bacterial groups present in both the soil sample and internal swabs: *Paracoccus*, *Cesiribacter* and *Psychrobacter* (including the species *Psychrobacter cryohalolentis*, previously isolated from the saline permafrost (Yair et al. 2021)). The *Cesiribacter* genus includes species previously isolated from desert and volcano soils (Xu et al. 2015). However, none of these genera are commonly associated with humans. Yair et al. (2021) performed a similar sequencing study during a Mars analogue mission in the Ramon crater, Israel. They reported the detection of known human‐associated taxa in the soil immediately outside the habitat airlock, and at much higher levels by their sanitary disposal tank, as evidence for forward contamination of the environment, but these were not specifically matched to a characterisation of the habitat microbiome.

Three eukaryotic ASVs were also common between our outdoor and indoor sample locations. Two sequences were identified as Ascomycota fungi: *Leptobacillium leptobactrum*, a fungus previously identified in low‐nutrient soil environments (Daghino et al. 2009); and *Aureobasidium pullulans*, a cosmopolitan fungal species. The third sequence was identified as *Triticum aestivum* —wheat—which potentially represents pollen that was dispersed to the Utah desert where the sampling was performed. These bacteria and fungi identified in the interior swap samples provide evidence of backwards contamination of environmental microbes being brought into the Hab during MDRS operations.

The absence of detectable forward contamination from the Hab into the exterior environment could be the result of multiple factors, including low shedding from the habitat (Schuerger and Lee 2015) and the use of simulated spacesuits outside the Hab reducing the transfer of microbes into the environment. To estimate the detection threshold for the molecular profiling techniques employed in this study, we need to consider each processing step: 20 g of soil was used for DNA extraction and sequenced to a depth of ~60,000 reads, which corresponds to 3000 reads per gram of soil. Assuming a reliable detection requires at least 10 reads, the minimum detectable bacterial population would be 3 million bacteria per gram of soil. If we consider a single read as sufficient for detection, the threshold decreases to approximately 333,000 bacteria per gram; however, this assumes that DNA extraction would be 100% efficient, and it is therefore fair to assume that even higher abundances would be required for detection. These estimates highlight the sensitivity limitations of our sequencing approach, and it is possible that a greater sequencing depth or use of a more sensitive technique (i.e., digital PCR) may be capable of identifying potential contamination present at lower levels. The detection of bacterial and eukaryotic ASVs shared between the internal and external environment, and their association with either soil or plants, would suggest that these sequences represent reverse contamination and the transfer of microbes from the external environment into the Hab.

## 4 Conclusions

One major concern regarding the future human exploration of Mars is the risk of contamination of the Martian surface by microorganisms from within the crew habitats. Such an eventuality could frustrate astrobiological efforts to identify indigenous Martian microbial life. Whilst robotic missions can be thoroughly cleansed to meet planetary protection protocols, astronauts cannot, and the microbiome of the inhabited environment is dominated by human‐associated microbes.

In this study of the MDRS analogue site, the dominant bacterial genera across the sampled interior surfaces were the Gram‐negative *Acinetobacter*, *Pseudomonas*, *Escherichia* and *Shigella*, many members of which are human commensals (and potential pathogens). Enteric bacteria were found in all indoor locations. Plant‐associated bacteria, such as *Pantoea*, dominated the top of the dining table, presumably from crew meal preparation. Plant DNA was also identified in the kitchen, including wheat, potato and tomato, and henna DNA was identified on the computer keyboard (attributed to a crewmember with dyed hands) further indicating the cross‐contamination of biological material within the Hab. The predominant fungal sequences detected in interior swabs were *Penicillium* and *Aureobasidium*, with the mycobiome of the EVA door handle found to be most similar to that of the soil sample. No archaeal sequences were identified in either interior swab samples or environmental soil. These results contribute to the growing literature on the characterisation of the human‐associated microbiome within crewed facilities in isolated locales, including space analogue missions, and highlight the necessity of monitoring the microbiome within such confined habitats.

DNA sequences amplified from the soil sample showed the environmental microbiome to have a great diversity of taxa that did not appear in the internal samples. This environmental sample was mostly characterised by Bacteroidota, Actinobacteriota and Pseudomonodata bacteria, with a number of psychrophilic, halotolerant and desiccation‐resistant extremophiles identified. The most abundant fungal genera were *Alternaria*, *Neocamarosporium* and *Preussia*. Principal Component Analysis of the 16S rRNA gene and ITS profiles between the soil sample and at least one indoor sample showed distinct clustering based on location, with this significant difference between indoor and outdoor samples confirmed via Kruskal Wallis, and thus providing no evidence for contamination of the soil from the Hab microbiome. Sequences belonging to the *Pseudomonas* genus, which contains common human commensals, were abundant in the internal swabs and also detected in the soil sample and so potentially represented evidence of forward contamination. This was dismissed, however, as at the ASV level the internal and external populations are distinct and not indicative of transference. However, three bacterial genera—*Paracoccus, Cesiribacter* and *Psychrobacter*—were identified in both soil and internal swabs and are not commonly associated with humans. These represent evidence of backwards contamination of environmental microbes being brought into the Hab during MDRS operations.

The absence of evidence detected here for the escape of human‐associated microbes from the MDRS Martian analogue facility into the immediate environment is not evidence that it does not happen. Such microbiome contamination could be occurring below the detection threshold of the sampling and sequencing approach adopted by this study. The evidence presented here of backwards contamination, however, does demonstrate the potential for microbial transference between crewed infrastructure and the surface environment. The MDRS is not a perfect simulation of the functioning of a future Martian habitat—with actual airlocks and crew EVAs within fully sealed, pressurised space suits—but these results do underscore the risk of microbial transference between such a facility and the Martian surface. This is a risk that must be fully considered and mitigated against to preserve the pristine nature of the Martian surface from terrestrial contamination, not least for the interests of astrobiology and the search for indigenous Martian microbial life.

## Author Contributions

**Mara Leite:** investigation, writing – original draft, methodology, writing – review and editing, formal analysis, project administration, visualization. **Dragana Dobrijevic:** writing – review and editing, methodology, formal analysis, investigation. **Michael C. Macey:** writing – review and editing, methodology, formal analysis, software, investigation, visualization. **Mamatha Maheshwarappa:** writing – review and editing, investigation. **John Ward:** writing – review and editing, supervision. **Lewis Dartnell:** conceptualization, investigation, writing – original draft, methodology, visualization, writing – review and editing, formal analysis, project administration, supervision, resources.

## Conflicts of Interest

The authors declare no conflicts of interest.

## Supporting information


**Data S1.** Supporting Information.

## Data Availability

The data that supports the findings of this study are available in the supplementary material of this article.
