# Supplementary material for: Analysis of Microbial Diversity and Evidence of Contamination at a Mars Analogue Habitat
Source: Environ Microbiol Rep. 2025 Jul 6;17(4):e70146. doi: 10.1111/1758-2229.70146 (PMC12229742; doi:10.1111/1758-2229.70146)
Supplement: Supplementary file 1 — Data S1. Supporting Information. [file EMI4-17-e70146-s001.docx]

**Supplementary Information (SI)**

Table SI 1. Bacterial genera labeled as contaminants based on the number of reads in each sample and the control

| Bacteria (genus) | Kitchen shelf | Fridge door | Dining table | Computer keyboard | Workshop door handle | EVA airlock handle | Soil | Control |
| --- | --- | --- | --- | --- | --- | --- | --- | --- |
| *Phyllobacterium* | 144 | 1251 | 8304 | 24903 | 994 | 73 | 0 | 49859 |
| *Bradyrhizobium* | 0 | 53 | 403 | 3306 | 525 | 36 | 0 | 3390 |
| *Mesorhizobium* | 0 | 0 | 21 | 511 | 20 | 0 | 11 | 5743 |
| *Labrys* | 0 | 0 | 63 | 488 | 207 | 0 | 0 | 582 |
| *Citrobacter* | 0 | 0 | 0 | 0 | 0 | 0 | 0 | 69 |

Table SI 2. Fungal genera labeled as contaminants based on the number of reads in each sample and the control

| Fungi (genus) | Kitchen shelf | Fridge door | Dining table | Computer keyboard | Staircase handrail | Workshop door handle | EVA airlock handle | Soil | Control |
| --- | --- | --- | --- | --- | --- | --- | --- | --- | --- |
| Leptobacillium | 52064 | 24736 | 97650 | 9789 | 43391 | 31630 | 90755 | 0 | 57596 |
| Exophiala | 13221 | 19670 | 48893 | 4897 | 43252 | 32269 | 59866 | 0 | 16286 |
| Malassezia | 1651 | 0 | 2089 | 5757 | 198 | 1683 | 7975 | 0 | 716 |
| Oidiodendron | 0 | 0 | 0 | 0 | 0 | 0 | 0 | 0 | 157 |
| Armillaria | 0 | 0 | 0 | 0 | 0 | 0 | 0 | 0 | 131 |

Table SI 3. Alpha diversity metrics for bacteria

|  | Shannon | Simpson | Chao1 |
| --- | --- | --- | --- |
| Kitchen shelf | 1.784884854 | 0.767955859 | 17 |
| Fridge door | 0.260726924 | 0.099798767 | 14 |
| Dining table | 1.099825516 | 0.481294241 | 35 |
| Computer keyboard | 2.433142581 | 0.80333838 | 65 |
| Workshop door handle | 0.380374298 | 0.157678289 | 29 |
| EVA airlock handle | 0.994337666 | 0.52617702 | 31 |
| Soil | 3.795881207 | 0.962518761 | 249 |
| Control | 0.840707393 | 0.379521089 | 17 |

Table SI 4. Alpha diversity metrics for fungi

|  | Shannon | Simpson | Chao1 |
| --- | --- | --- | --- |
| Kitchen shelf | 1.352421311 | 0.657347041 | 8 |
| Fridge door | 1.803409228 | 0.768215773 | 15 |
| Dining table | 1.108639698 | 0.558373867 | 12 |
| Computer keyboard | 1.796380453 | 0.764026415 | 14 |
| Staircase handrail | 1.399965216 | 0.679415886 | 9 |
| Workshop door handle | 1.913607283 | 0.792312552 | 12 |
| EVA airlock handle | 2.18072225 | 0.849087383 | 18 |
| Soil | 1.935061367 | 0.736013432 | 58 |


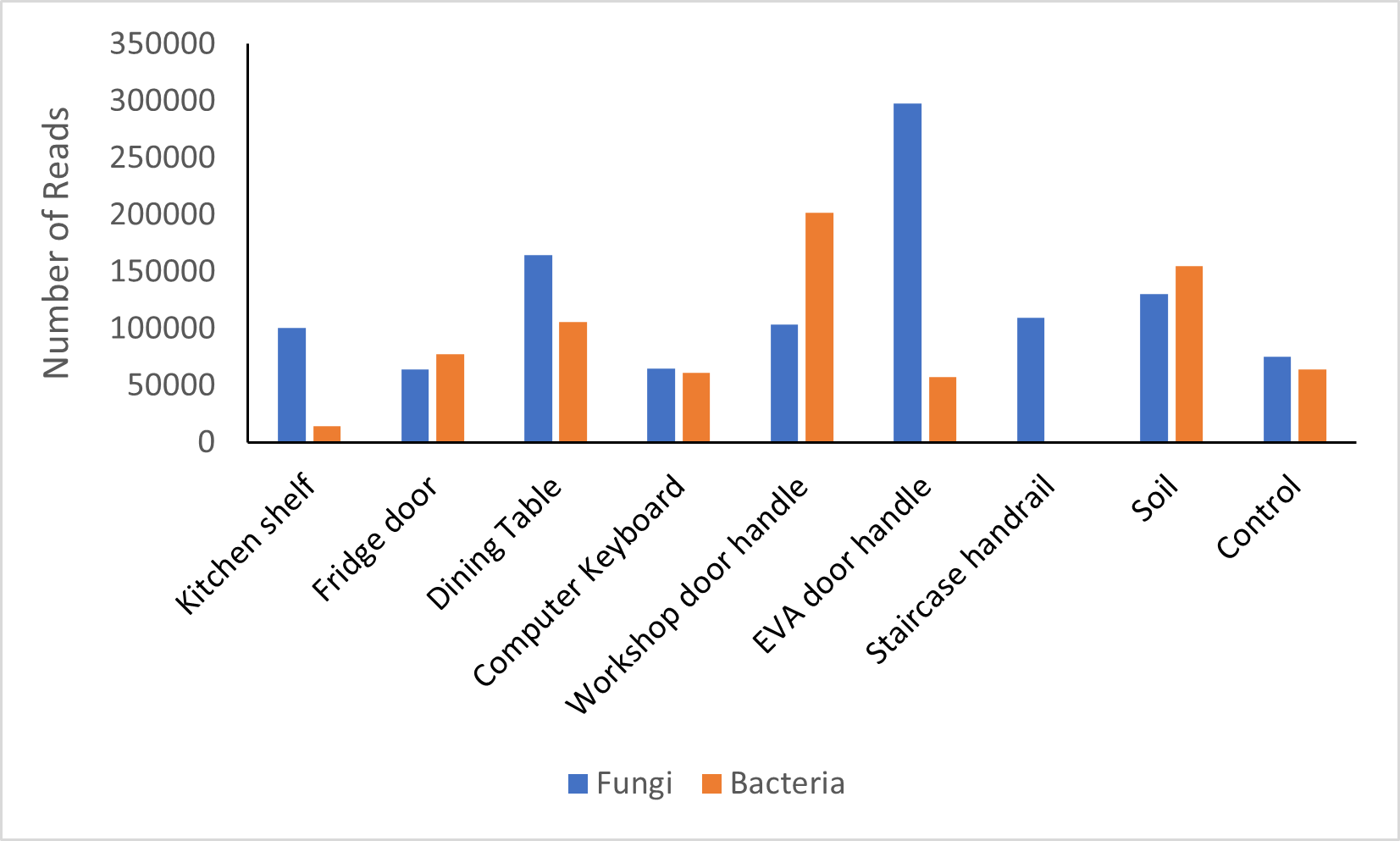


Figure SI 1. Number of reads in all samples for both Bacteria and Fungi.


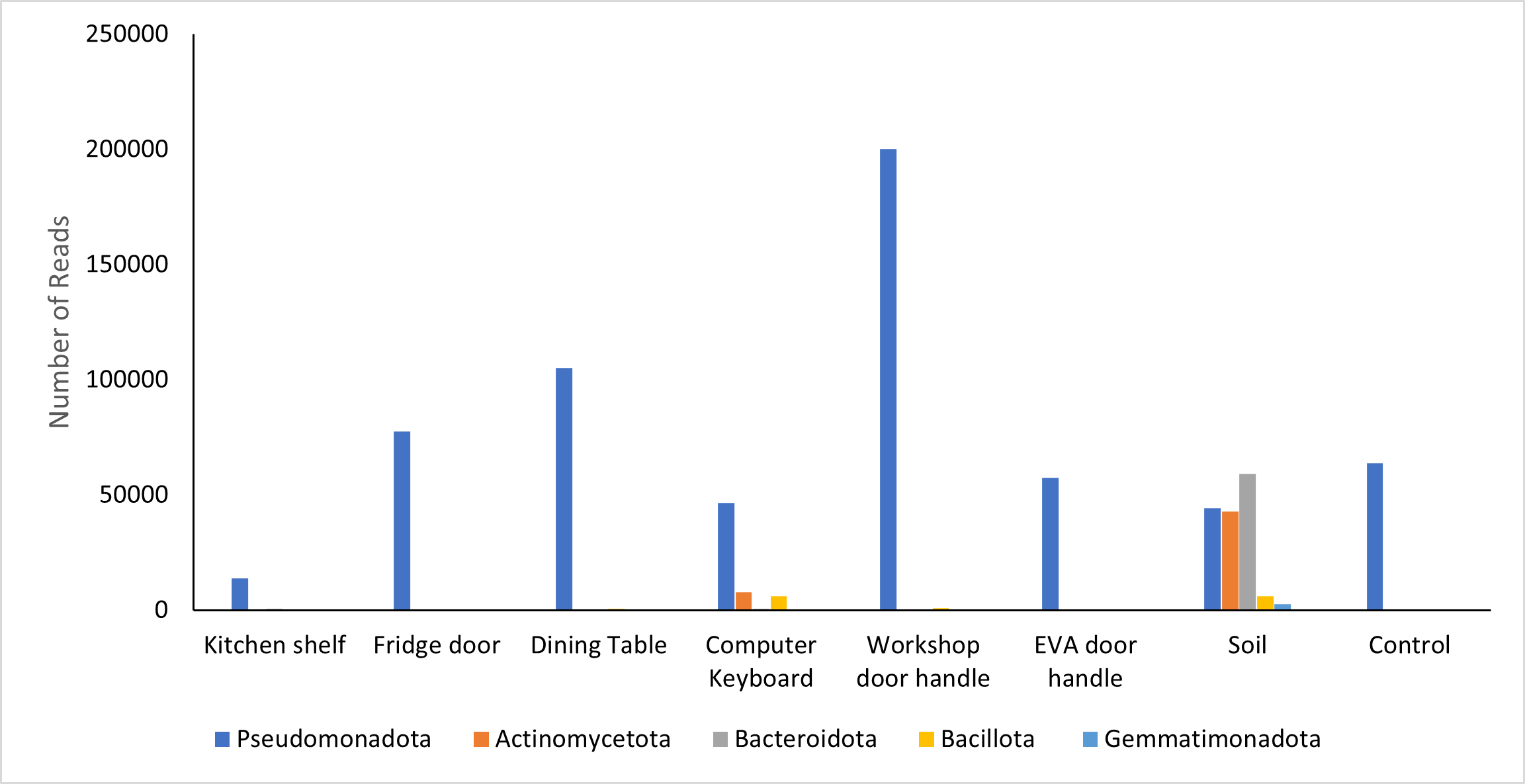


Figure SI 2. Predominant bacterial phyla in each sample.


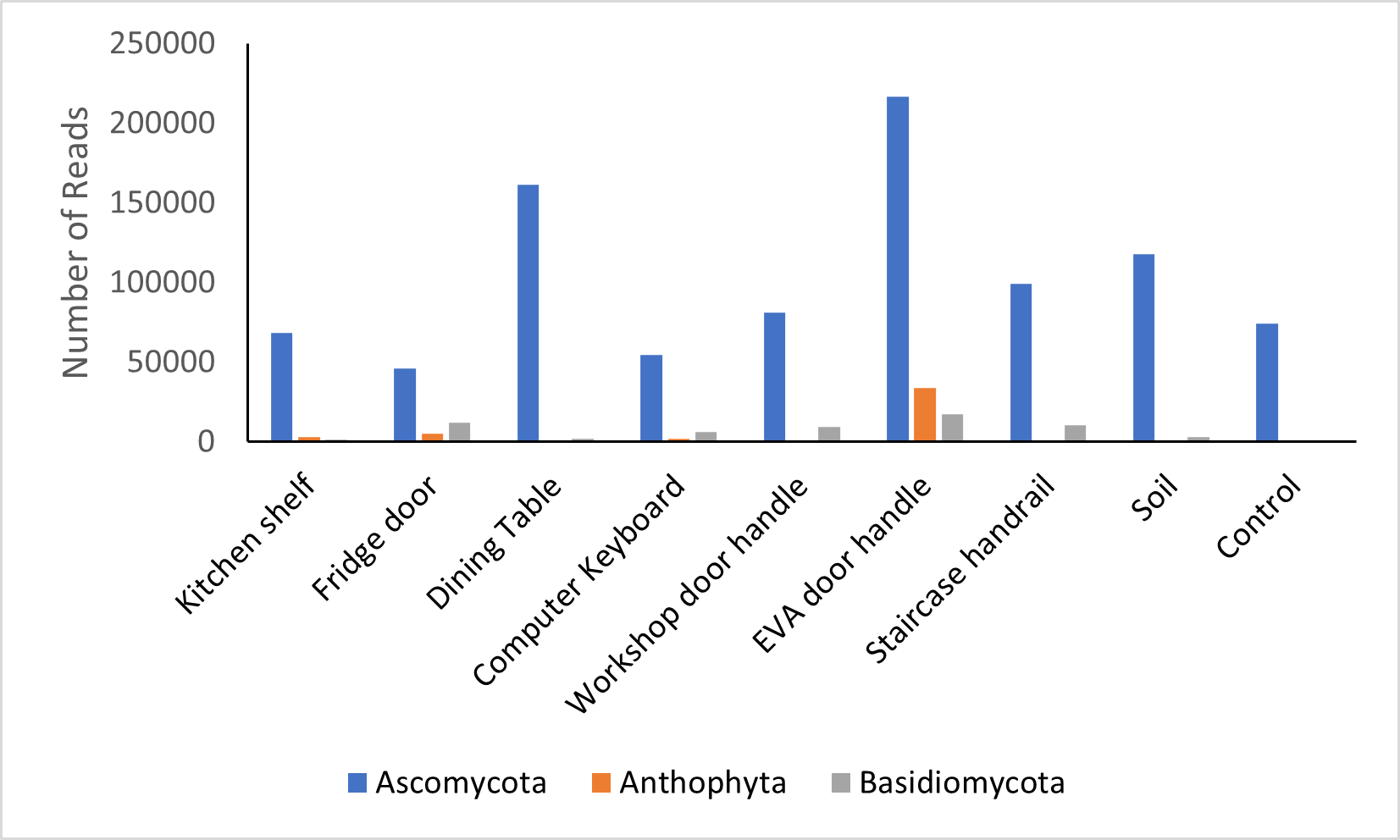


Figure SI 3. Predominant fungal phyla in each sample.

Graphical Abstract

We assessed the potential of a crewed mission to Mars to microbially contaminate the surface by studying a terrestrial analogue facility, the Mars Desert Research Station. DNA sequencing of interior swabs and an external soil sample allowed us to characterize the microbiomes present. We didn’t detect contamination of the desert soil within our detection thresholds, but did find evidence of backwards contamination of environmental microbes into the MDRS.
